# Impact of concomitant cholecystectomy during gastrectomy for gastric cancer on the postoperative quality of life

**DOI:** 10.1007/s00595-026-03294-0

**Published:** 2026-04-07

**Authors:** Masataka Shimonosono, Takaaki Arigami, Keishi Okubo, Daisuke Matsushita, Masahiro Noda, Yusuke Tsuruda, Ken Sasaki, Kenji Baba, Takao Ohtsuka

**Affiliations:** Department of Digestive Surgery, Kagoshima University Graduate School of Medical and Dental Sciences, 8-35-1 Sakuragaoka, Kagoshima, 890-8520 Japan

**Keywords:** Gastrectomy, Cholecystectomy, Diarrhea, Quality of life, Gastric cancer

## Abstract

**Purpose:**

This study aimed to evaluate the impact of concomitant prophylactic cholecystectomy during gastrectomy for gastric cancer on the postoperative quality of life (QOL) and nutritional outcomes.

**Methods:**

A retrospective analysis was conducted on 318 patients who underwent curative gastrectomy for gastric cancer between 2010 and 2023. Twenty-eight patients underwent prophylactic cholecystectomy, while 290 underwent gastrectomy alone. The postoperative QOL at one year was assessed using the Postgastrectomy Syndrome Assessment Scale-45, and the nutritional status was evaluated using body weight change.

**Results:**

The operative time was significantly longer in the cholecystectomy group. However, blood loss and major complications were comparable. There was no significant difference between the two groups in the postoperative cholelithiasis-free rate or cholecystitis/cholangitis-free rate. One year postoperatively, the body weight loss was similar; however, diarrhea-related symptom scores were significantly higher in the cholecystectomy group (3.0 vs. 1.7, *p* = 0.01). A multivariate analysis identified male sex and concomitant cholecystectomy as independent risk factors for postoperative diarrhea.

**Conclusion:**

Prophylactic cholecystectomy during gastrectomy did not increase major complications, but it was associated with worse diarrhea-related symptoms and a lower QOL. Its indications should therefore be carefully considered, while balancing the potential benefits against the long-term functional outcomes.

## Introduction

Transection of the vagus nerve during gastrectomy is known to impair gallbladder contraction, resulting in reduced bile excretion. This leads to prolonged bile stasis within the gallbladder, thereby promoting gallstone formation [1, 2]. For asymptomatic cholelithiasis, bservation is generally recommended, with surgical intervention reserved for symptomatic cases [3]. However, due to the increased risk of gallstone-related complications, such as biliary colic or cholecystitis, following gastrectomy, prophylactic cholecystectomy is often performed concurrently [4, 5].

The reported incidence of cholelithiasis after gastrectomy for gastric cancer ranges from 5.2% to 12.3%, while the proportion of patients requiring treatment for symptomatic cholelithiasis is reported to be 0.6% to 4.6% [4, 6–8]. A randomized controlled trial found no significant difference in the cholelithiasis-free survival between the patients undergoing prophylactic cholecystectomy and those undergoing gastrectomy alone, thus suggesting that the benefit of prophylactic cholecystectomy remains controversial [8]. Conversely, a meta-analysis found no significant increase in the postoperative complications associated with prophylactic cholecystectomy [9]. Moreover, delayed cholecystectomy after gastrectomy has been associated with a higher incidence of postoperative complications than standard cholecystectomy [10], highlighting the potential benefit of avoiding reoperation.

Few studies have so far addressed the potential disadvantages of prophylactic cholecystectomy. Postcholecystectomy syndrome, a constellation of symptoms including persistent right upper quadrant pain, abdominal distension, diarrhea, and nausea, has been reported in 5%–47% of patients following cholecystectomy [11]. These symptoms may negatively impact the postoperative quality of life (QOL). However, to date, no studies have evaluated the effect of prophylactic cholecystectomy on QOL using validated patient-reported outcome measures in the context of gastrectomy.

In this study, we retrospectively analyzed the postoperative quality of life (QOL) one year after gastrectomy in patients with gastric cancer using patient-reported outcome measures. We aimed to evaluate the impact of concomitant prophylactic cholecystectomy on the long-term postoperative QOL.

## Methods

### Patients

**Fig. 1:**
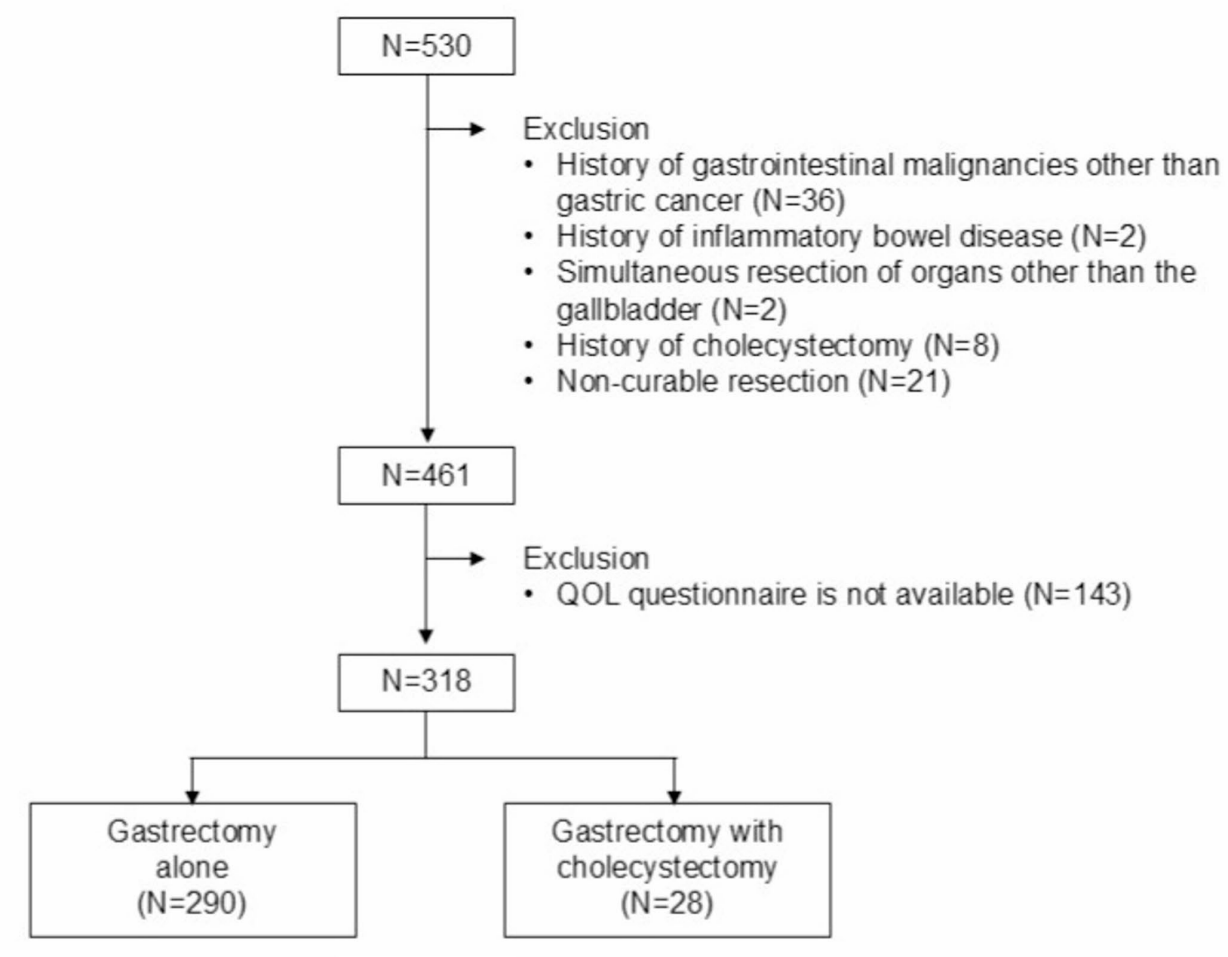
Patient selection flowchart. A total of 530 patients with gastric cancer who underwent curative gastrectomy between January 2010 and March 2023 were screened. After applying exclusion criteria and removing cases without one-year postoperative quality of life data, 318 patients were included in the final analysis. Of these, 290 underwent gastrectomy alone, and 28 underwent gastrectomy with concomitant prophylactic cholecystectomy

We retrospectively enrolled 530 patients with gastric cancer who underwent curative gastrectomy at Kagoshima University between January 2010 and March 2023. Patients were excluded if they had a history of gastrointestinal malignancies other than gastric cancer, inflammatory bowel disease, simultaneous resection of organs other than the gallbladder, prior cholecystectomy, or non-curative resection. These criteria led to the exclusion of 69 patients. An additional 143 patients were excluded due to missing quality of life (QOL) survey data at one year postoperatively, resulting in a final cohort of 318 patients. Asymptomatic cholelithiasis was defined as the presence of radiopaque gallstones on computed tomography (CT) without gallstone-related symptoms. No patient had symptomatic cholelithiasis preoperatively. Patients with asymptomatic cholelithiasis at the time of gastric cancer diagnosis were considered eligible for prophylactic cholecystectomy. Among the 318 patients included in the final analysis, 290 underwent gastrectomy alone, whereas 28 underwent gastrectomy with concomitant prophylactic cholecystectomy (Fig. 1). This study was approved by the Institutional Review Board of Kagoshima University (Approval No. 230068).

### Treatment

The type of gastrectomy was determined according to the location and stage of gastric cancer. For lesions located distal to the middle third of the stomach, distal gastrectomy (DG) was performed; Billroth-I reconstruction (B-I) was the first choice, and Roux-en-Y (RY) or Billroth-II reconstruction (B-II) was selected when B-I was not feasible due to a small remnant stomach. Proximal gastrectomy (PG) was performed for early-stage cancers confined to the esophagogastric junction or the upper third of the stomach; esophagogastrostomy (EG) was chosen when the remnant stomach was ≥ two-thirds in size and there was no esophageal invasion, whereas double-tract reconstruction (DT) was selected in other cases. In all other cases, total gastrectomy (TG) with RY was performed. Regarding the vagus nerve, the hepatic branch was preserved and the celiac branch was divided in DG, whereas both branches were divided in PG and TG. In patients diagnosed with cholelithiasis preoperatively, cholecystectomy was performed consecutively after gastrectomy. Postoperative follow-up was conducted at our hospital or affiliated local clinics; in principle, computed tomography (CT) examinations were performed every six months until three years after surgery and then annually thereafter up to five years.

### Outcome measures

The nutritional status was assessed by calculating the percentage change in body weight at 1, 3, and 5 years postoperatively compared with the preoperative baseline. Postoperative QOL was evaluated using the Postgastrectomy Syndrome Assessment Scale-45 (PGSAS-45), as previously described [12–14]. The PGSAS-45 questionnaire comprises 45 items: eight derived from the Short Form Health Survey-8, 15 from the Gastrointestinal Symptoms Rating Scale, and 22 originally selected by the Japan Postgastrectomy Syndrome Working Party. Each item is rated on a five- or seven-point Likert scale and covers domains including postoperative symptoms, living status, and overall QOL. Seven main outcome measures (MOMs) assessed postoperative symptoms: esophageal reflux, indigestion, abdominal pain, dumping syndrome, diarrhea, constipation, and meal-related distress. Three MOMs evaluated living status: quality of ingestion, amount of food per meal, and necessity for additional meals. Four MOMs assessed QOL: dissatisfaction with symptoms, meals, work, and daily life. The questionnaire was distributed directly to patients when they visited the outpatient clinic for their 1-year postoperative follow-up and was completed and collected without face-to-face interaction with a physician.

### Statistical analysis

Group comparisons were performed using Student’s *t*-test, the chi-square test, or Fisher’s exact test, as appropriate. A linear regression analysis was used to assess the relationships between continuous variables. A multivariate analysis of risk factors was performed using a multiple regression analysis. The cholelithiasis-free rate and cholecystitis/cholangitis-free rate were measured from the day of surgery and calculated using the Kaplan–Meier method. Differences in the incidence between groups were evaluated using the log-rank test. All statistical analyses were conducted using JMP Pro 16 (SAS Institute, Cary, NC, USA). Statistical significance was considered to be a *p*-value of < 0.05.

## Results

### Patient characteristics

QOL assessment was not conducted in 143 patients who did not receive a postoperative follow-up at our institution because of factors such as advanced age, a poor performance status (PS), residence outside the city, or recurrence/death within one year after surgery. Compared with patients who underwent the QOL assessment, those without an assessment had significantly higher rates of age ≥ 80 years (26% vs. 11%), PS ≥ 1 (31% vs. 12%), residence outside the city (68% vs. 49%), pStage ≥ II disease (48% vs. 30%), and relapse-free survival (RFS) < 365 days (22% vs. 2%) (all *p* < 0.001) (Online Resource 1).

Of the 318 patients included in the final analysis, 290 were assigned to the gastrectomy-alone group and 28 to the gastrectomy with cholecystectomy group. No significant differences were observed between the two groups with respect to age, sex, preoperative PS, serum albumin levels, surgical procedure, reconstruction method, pathological stage, or adjuvant chemotherapy. The regimen and duration of adjuvant chemotherapy, as well as the use of oral anticancer agents at one year postoperatively, were also comparable between the two groups. Furthermore, there was no significant difference between the groups in the status of bowel regulator use (including probiotics, antidiarrheals, and laxatives) at one year postoperatively. However, the preoperative body mass index (BMI) was significantly higher in the concomitant cholecystectomy group (median 24.3, range 16.9–33.3) than in the gastrectomy-alone group (median 22.7, range 14.7–41.2; *p* = 0.003) (Table 1).

**Table 1:** Patient characteristics

Factor	Gatrectomy alone (*N*=290)	Gastrectomy with cholecystectomy (*N*=28)	*p*-value
Sex, male, n (%)	215 (74)	22 (79)	0.82
Age (years), median (range)	69 (19–91)	69 (50–81)	0.98
Performance status, 0/1/2, n (%)	256/29/4 (89/10/1)	22/6/0 (79/21)	0.17
Body mass index, median (range)	22.7 (14.7–41.2)	24.3 (16.9–33.3)	0.003
Alb (g/dl), median (range)	4.1 (2.8–5.3)	4.1 (2.7–4.8)	0.56
Surgical procedure, n (%)			0.79
DG, Billroth I	106 (37)	11 (39)	
DG, Roux-en-Y or Billroth II	68 (23)	9 (32)	
PG, Double-tract	44 (15)	3 (11)	
PG, Esophagogastric anastomosis	17 (6)	1 (4)	
TG, Roux-en-Y	55 (19)	4 (14)	
Duodenal exclusion, yes, n (%)	123 (42)	13 (46)	0.69
Histological type, diffuse, n (%)	177 (61)	13 (46)	0.15
Postoperative diagnosis			
Tumor depth, pT1/T2-4, n (%)	175/115 (61/39)	20/8 (71/29)	0.31
Nodal metastasis, pN+, n (%)	240/50 (83/17)	21/7 (75/25)	0.31
Distant metastasis, pM1, n (%)	0 (0)	0 (0)	1.00
pStage, 0,I, II, III, n (%)	7/194/53/36 (2/67/18/13)	0/19/3/6 (0/68/11/21)	0.45
Adjuvant chemotherapy, n (%)			0.83
S1	37 (13)	6 (21)	
Docetaxel+S1	25 (9)	2 (7)	
Docetaxel	3 (1)	0 (0)	
S1+Paclitaxel ip/iv	3 (1)	0 (0)	
S1+oxaliplatin	3 (1)	0 (0)	
Others	6 (2)	0 (0)	
No	213 (73)	20 (72)	
Duration of adjuvant chemotherapy (month), median (range)	12 (2-60)	12 (6-43)	0.37
Use of oral chemotherapy at one year after surgery, yes, n (%)	49 (17)	6 (21)	0.54
Use of bowel regulators at one year after surgery, yes, n (%) *			
Probiotics	33 (12.8)	2 (7.4)	0.55
Antidiarrheals	3 (1.2)	1 (3.7)	0.33
Laxatives	57 (22.2)	5 (18.5)	0.81

*Information regarding the use of bowel regulators was unavailable for 34 patients (10.7%). DG: distal gastrectomy; PG: proximal gastrectomy; TG: total gastrectomy; ip: intraperitoneal administration; iv: intravenous administration

### Surgical outcome

The operative time was significantly longer in the concomitant cholecystectomy group (median 476 min, range, 340–712) than in the gastrectomy-alone group (median 400 min, range, 185–731; *p* < 0.001). No significant differences were found between the groups in terms of intraoperative blood loss or the incidence of postoperative complications classified as Clavien–Dindo grade ≥ 3 (Table 2).

**Table 2:** Surgical outcomes

Factor	Gatrectomy alone (*N*=290)	Gastrectomy with cholecystectomy (*N*=28)	*p*-value
Operating time (min), median (range)	400 (185–731)	476 (340–712)	<0.001
Blood loss (ml), median (range)	78 (0–2250)	97 (0–1350)	0.99
Clavien-Dindo, grade ≥3, n (%)	24 (8)	3 (11)	0.59

### Risk of cholelithiasis and cholecystitis/cholangitis after gastrectomy

**Fig. 2:**
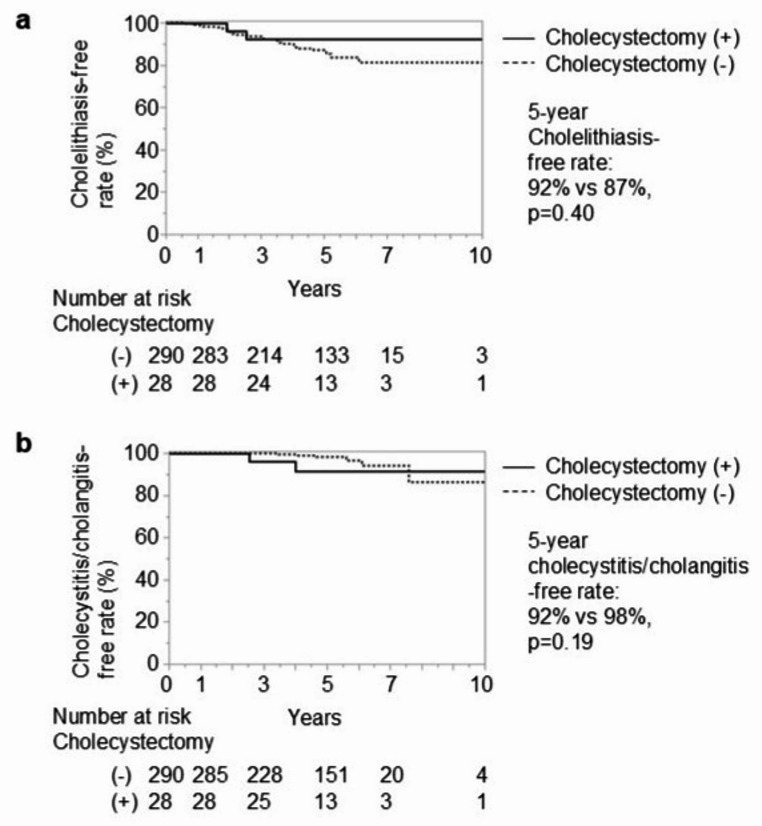
Cholelithiasis-free rate and cholecystitis/cholangitis-free rate after gastrectomy. The 5-year cholelithiasis-free rate were 92% in the concomitant cholecystectomy group and 87% in the gastrectomyalone group (**a**). The 5-year cholecystitis/cholangitis-free rate were 92% in the concomitant cholecystectomy group and 98% in the gastrectomy-alone group (**b**). There were no significant differences between the two groups in cholelithiasis-free rate (*p* = 0.40) or cholecystitis/cholangitis-free rate (*p* = 0.19)

In the gastrectomy-alone group, the 5-year cholelithiasis-free rate and cholecystitis/cholangitis-free rate were 87% and 98%, respectively. In contrast, in the concomitant cholecystectomy group, two of 28 patients developed cholangitis due to common bile duct stones and underwent endoscopic treatment; the 5-year cholelithiasis-free rate and cholecystitis/cholangitis-free rate were 92% and 92%, respectively. There were no significant differences between the two groups in the cholelithiasis-free rate (*p* = 0.40) or the cholecystitis/cholangitis-free rate (*p* = 0.19) (Fig. 2).

In the analysis stratified by surgical procedure within the gastrectomy-alone group, the incidence of cholelithiasis was 6.6% (7/108) in the DG/B-I group, 8.8% (6/68) in the DG/RY or B-II group, 15.9% (6/44) in the PG/DT group, 5.9% (1/17) in the PG/EG group, and 21.8% (12/55) in the TG/RY group. The incidence of cholecystitis/cholangitis was 0.9% (1/108) in the DG/B-I group, 1.5% (1/68) in the DG/RY or B-II group, 2.3% (1/44) in the PG/DT group, 0% (0/17) in the PG/EG group, and 5.5% (3/55) in the TG/RY group. The proportion of patients with cholelithiasis who subsequently developed cholecystitis/cholangitis was 14.3% (1/7) in the DG/B-I group, 16.7% (1/6) in the DG/RY or B-II group, 16.7% (1/6) in the PG/DT group, and 25% (3/12) in the TG/RY group. In the concomitant cholecystectomy group, the incidence of cholelithiasis was 9.1% (1/11) in the DG/B-I group and 25% (1/4) in the TG/RY group; similarly, the incidence of cholecystitis/cholangitis was 9.1% (1/11) in the DG/B-I group and 25% (1/4) in the TG/RY group (Online Resource 2).

### Postoperative nutritional status and quality of life

**Fig. 3:**
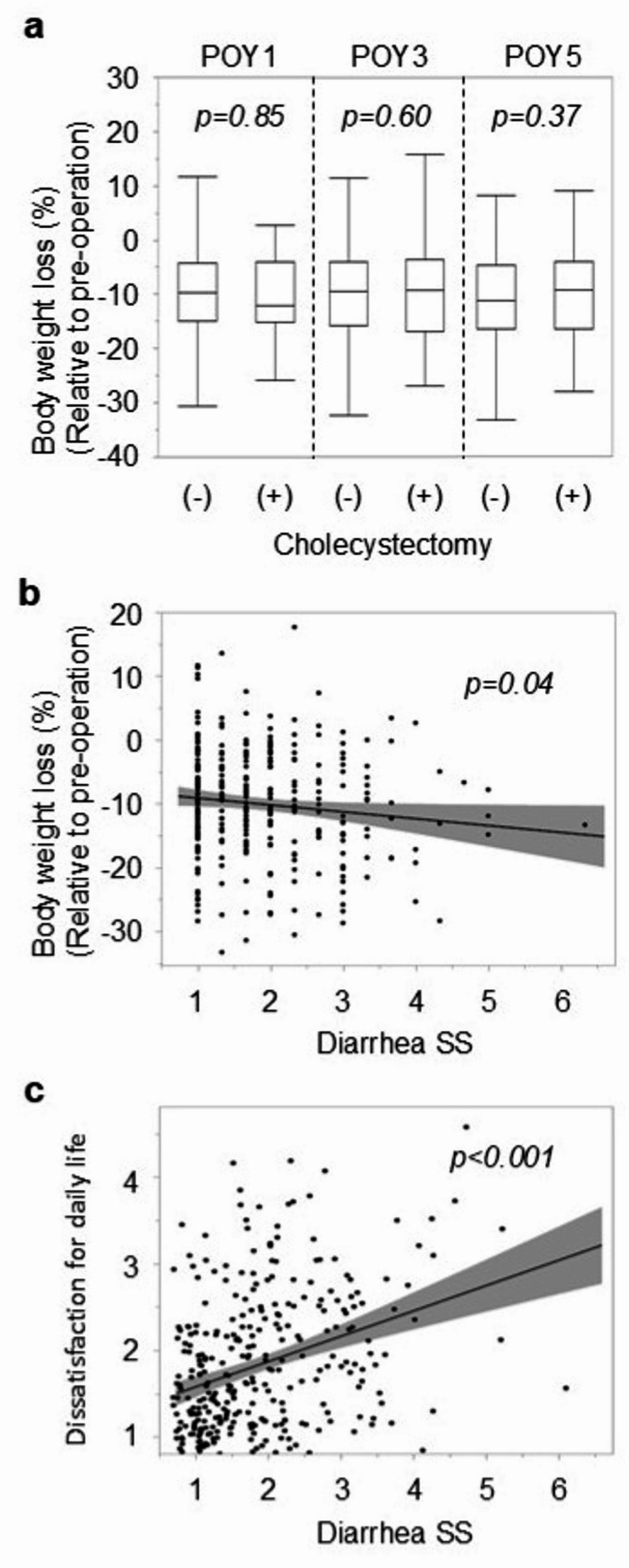
Postoperative Nutritional Status and Quality of Life. (**a**) The percentage change in body weight at 1, 3, and 5 years after surgery did not differ between patients who underwent gastrectomy alone and those who underwent gastrectomy with concomitant prophylactic cholecystectomy (*p* = 0.85, *p* = 0.6 and *p* = 0.37). (**b**) The diarrhea-related symptom score showed a significant association with the extent of postoperative weight loss (β = −0.12, 95% CI −2.08 to −0.07, *p* = 0.04). (**c**) Diarrhea-related symptom scale scores were significantly associated with dissatisfaction for daily life (β = 0.33, 95% CI 0.2 to 0.38, *p* < 0.001). POY: postoperative year

At 1, 3, and 5 years postoperatively, the change in body weight was comparable between the two groups (Fig. 3a). However, the diarrhea-related symptom score was significantly higher in the concomitant cholecystectomy group (mean 3.0, standard deviation [SD] 1.3) than in the gastrectomy-alone group (mean 1.7, SD 0.9; Cohen’s *d* = 0.76, *p* = 0.01). No significant differences were observed in other symptom scales related to postgastrectomy symptoms (Table 3). Likewise, no significant differences were found in the subscales assessing the living status or dissatisfaction with symptoms, meals, work, or daily life (Table 3).

**Table 3:** Assessment of postoperative symptoms based on PGSAS-45

Factor		Gatrectomy alone	Gastrectomy with cholecystectomy	Cohen’s d	*p*-value
		Mean (SD)	Mean (SD)		
Postoperative symptoms					
	Esophageal reflux SS	1.5 (0.8)	1.4 (0.6)	-0.16	0.31
	Indigestion SS	1.8 (0.8)	1.8 (0.8)	-0.01	0.97
	Abdominal pain SS	1.3 (0.7)	1.3 (0.6)	-0.05	0.77
	Dumping SS	1.3 (1.1)	1.3 (1.0)	0.02	0.91
	Diarrhea SS	1.7 (0.9)	3.0 (1.3)	0.76	0.01
	Constipation SS	2.0 (1.1)	2.0 (0.8)	-0.01	0.95
	Meal-related distress SS	2.0 (0.8)	2.0 (0.9)	-0.06	0.77
Living status					
	Quality of ingestion SS	3.7 (1.0)	4.0 (1.1)	0.11	0.62
	Ingested amount of food	7.0 (2.0)	7.0 (2.1)	-0.07	0.75
	Necessity for additional meals	2.0 (0.8)	2.0 (0.9)	-0.27	0.20
Quality of life					
	Dissatisfaction with symptoms	1.0 (0.8)	1.0 (0.7)	-0.28	0.12
	Dissatisfaction at the meal	2.0 (1.1)	2.0 (1.0)	-0.24	0.19
	Dissatisfaction at working	1.0 (0.9)	1.0 (0.8)	-0.12	0.52
	Dissatisfaction for daily life	1.7 (0.8)	1.7 (0.7)	-0.24	0.15

PGSAS-45: Postgastrectomy Syndrome Assessment Scale-45, SD: standard deviation, SS: subscale

The diarrhea-related symptom score was significantly associated with the extent of postoperative weight loss (β = −0.12, 95% CI − 2.08 to − 0.07, *p* = 0.04) (Fig. 3b). Furthermore, diarrhea-related symptom scores were significantly associated with dissatisfaction scores related to symptoms, meals, work, and daily life (β = 0.33, 95% CI 0.2 to 0.38, *p* < 0.001) (Fig. 3c).

### Risk factors for postoperative diarrhea

A multiple regression analysis was performed to identify the factors associated with postoperative diarrhea. No significant associations were found with age, body mass index (BMI), surgical procedure, reconstruction method, pathological factors, or use of postoperative adjuvant chemotherapy. However, being a male (β = 0.13, 95% confidence interval [CI] 0.02–0.26, *p* = 0.02) and concomitant prophylactic cholecystectomy (β = 0.2, 95% CI 0.14–0.5, *p* < 0.001) were identified as independent risk factors for postoperative diarrhea (Table 4).

**Table 4:** A multiple regression analysis for the risk factors associated with postoperative diarrhea

	β (95% CI)	*p*-value
Age	−0.07 (−0.01 to 0.004)	0.24
Sex, Male	0.13 (0.02 to 0.26)	0.02
Body mass index	0.06 (−0.01 to 0.04)	0.29
Total gastrectomy, Yes	0.02 (−0.13 to 0.19)	0.73
Duodenal exclusion, Yes	−0.007 (−0.13 to 0.12)	0.91
Cholecystectomy, Yes	0.2 (0.14 to 0.5)	<0.001
Histological type, Diffuse type	−0.06 (−0.17 to 0.05)	0.27
Pathological tumor depth, ≥T2	0.08 (−0.06 to 0.21)	0.27
Pathological nodal metastasis, N+	0.02 (−0.12 to 0.16)	0.77
Adjuvant chemotherapy, Yes	−0.09 (−0.25 to 0.06)	0.22

CI: confidence interval

## Discussion

Postcholecystectomy diarrhea (PCD) is a well-recognized complication following cholecystectomy. After gallbladder removal, bile is no longer concentrated and flows continuously into the intestine, leading to increased enterohepatic circulation of bile acids, enhanced intestinal motility, and shortened intestinal transit time, all of which contribute to diarrhea [15]. The reported incidence of PCD ranges from 2.1% to 57.2%, and it can significantly impair the postoperative quality of life [16].

Diarrhea is also a known component of post-gastrectomy syndrome, with an incidence ranging from 3.4% to 6.0% following gastrectomy [17, 18]. A younger age, male sex, total gastrectomy, and division of the celiac branch of the vagus nerve have been reported to be risk factors for diarrhea after gastrectomy [19]. In our cohort, a male sex and concomitant cholecystectomy were identified as independent risk factors for postoperative diarrhea, whereas no significant association was observed with age or type of gastrectomy. Kim et al. previously reported that concomitant cholecystectomy does not adversely affect the nutritional status following gastrectomy for gastric cancer [20], which is consistent with our findings. However, in the present study, postoperative diarrhea was significantly associated with postoperative weight loss and decreased QOL. Similarly, Nakada et al. reported a significant association between postoperative diarrhea and both weight loss and reduced QOL [19]. These results suggest that postoperative diarrhea should be considered a functional complication to be avoided whenever possible.

In the general population, the progression rate of asymptomatic cholelithiasis to symptomatic disease requiring surgery has been reported to be 10–18% at 5 years and 15–21.5% at 10 years [21–23]. In contrast, among the patients with asymptomatic cholelithiasis who underwent gastrectomy for gastric cancer, 30.8% required cholecystectomy within 5 years, indicating a higher risk of symptomatic cholelithiasis post-gastrectomy [24]. The risk of cholelithiasis after gastrectomy varies depending on the surgical procedure and reconstruction method. A meta-analysis reported a higher risk of cholelithiasis following total gastrectomy (7.7%) than after other types of gastrectomy (5.7%; OR 1.5, 95% CI 1.25–1.81) [9]. The procedures involving duodenal exclusion were associated with an 8.2% risk of cholelithiasis compared with 5.8% for those without duodenal exclusion (OR 1.77, 95% CI 1.47–2.15) [9]. Additionally, among the patients with cholelithiasis at the time of gastrectomy, 31.3% of those undergoing total gastrectomy and 7.4% of those undergoing distal gastrectomy without duodenal exclusion subsequently required cholecystectomy [23]. Other reported risk factors for post-gastrectomy cholelithiasis include an older age, male sex, and diabetes mellitus [9].

In the present study, the overall incidence of cholelithiasis and cholecystitis/cholangitis during the entire observation period in the gastrectomy-alone group was 11.4% and 2.1%, respectively, which was comparable to rates reported previously [4, 6–8]. In contrast, even in the concomitant cholecystectomy group, two patients (7.1%) developed postoperative cholangitis due to common bile duct stones, suggesting that the preventive effect of cholecystectomy on cholecystitis/cholangitis may be limited. Consistent with previous reports, the incidences of cholelithiasis and cholecystitis/cholangitis in the TG/RY subgroup after gastrectomy alone were 21.8% and 5.5%, respectively, which were higher than those in the DG/B-I subgroup (6.6% and 0%, respectively), indicating a potentially increased risk of gallstone-related events after TG. The incidences of cholelithiasis and cholecystitis/cholangitis in the DG/RY or B-II subgroup were 8.8% and 1.5%, respectively, which were lower than those in the TG/RY subgroup. These findings suggest that preservation of the hepatic branch of the vagus nerve may play an important role in preventing postoperative cholelithiasis after gastrectomy.

Simultaneous gastrectomy and cholecystectomy has been reported to be safe, without increasing postoperative complications compared with gastrectomy alone [9]. In contrast, secondary cholecystectomy after gastrectomy has been reported to be associated with a longer operative time and a greater blood loss compared with conventional cholecystectomy [10], thus supporting the rationale for prophylactic cholecystectomy to avoid reoperation. However, the primary factors compromising the safety of secondary cholecystectomy have been reported to be intra-abdominal and pericholecystic adhesions [10]. In these previous reports, the indications for gastrectomy included not only gastric cancer, but also gastroduodenal ulcer disease or perforation, and a substantial proportion of procedures were performed via an open approach. When limited to contemporary minimally invasive gastrectomy for gastric cancer, which has become the standard in recent years, postoperative intra-abdominal and pericholecystic adhesions are likely to be less severe; consequently, the surgical risk of secondary cholecystectomy may be lower. On the other hand, based on the results of the present study, the potential impact of persistent postoperative diarrhea on QOL should not be underestimated. Therefore, the indications for prophylactic cholecystectomy should be carefully weighed against the risk of developing symptomatic cholelithiasis after gastrectomy.

This study has several limitations. First, prophylactic cholecystectomy was performed only in patients with asymptomatic cholelithiasis, which may have introduced some selection bias. Thus, a potential association between the presence of cholelithiasis and postoperative diarrhea cannot be excluded. In the present study, the baseline characteristics were comparable between the two groups, except that the preoperative body mass index (BMI) was significantly higher in the concomitant cholecystectomy group. However, a multivariate analysis showed no significant association between a higher preoperative BMI and postoperative diarrhea. Second, there were limitations in assessing the use of bowel regulators, which are important factors for postoperative bowel symptoms. We could not capture the medication status for the entire cohort (data availability: 89.3%), and the potential use of over-the-counter drugs or dietary supplements was not fully recorded. Additionally, as many patients were treated by their primary care physicians, the indications for these prescriptions were not standardized. Despite these constraints, the available data showed no significant difference between the two groups in the use of bowel regulators. We believe that the inclusion of available medication data provides a more comprehensive understanding of the results of this study. Third, there are limitations regarding the imaging modalities used for the diagnosis of cholelithiasis. Ultrasonography is considered the gold standard for diagnosing cholelithiasis, with reported sensitivity and specificity of 95–99% and 88–100%, respectively. In contrast, computed tomography (CT) may fail to detect radiolucent gallstones, and its sensitivity and specificity have been reported to be 79% and 99%, respectively [25]. In this study, the diagnosis of gallstones was based solely on CT findings, which may have resulted in an underestimation of the prevalence of asymptomatic cholelithiasis. However, because preoperative ultrasonography was not routinely performed for all patients at our institution, the CT findings were used to ensure diagnostic consistency across the study population. Finally, this was a single-center, retrospective study with a relatively small sample size. Although the proportion of patients who underwent prophylactic cholecystectomy was relatively low (8.8% of the entire cohort), this percentage is considered acceptable because previous studies have reported that the prevalence of cholelithiasis in the general population ranges from 6.1% to 15% [26]. Future validation through large-scale, multicenter, prospective trials is warranted. Nevertheless, to our knowledge, no previous studies have evaluated the postoperative quality of life (QOL) using patient-reported outcome measures in the context of prophylactic cholecystectomy during gastrectomy.

## Conclusions

Prophylactic cholecystectomy significantly increased the risk of postoperative diarrhea and it may adversely affect the long-term postoperative quality of life. These findings therefore provide important insights to guide surgical decision-making in patients with gastric cancer.
